# Drug Repurposing Is a New Opportunity for Developing Drugs against Neuropsychiatric Disorders

**DOI:** 10.1155/2016/6378137

**Published:** 2016-03-17

**Authors:** Hyeong-Min Lee, Yuna Kim

**Affiliations:** ^1^Department of Cell Biology & Physiology, School of Medicine, University of North Carolina, 115 Mason Farm Road, Chapel Hill, NC 27599, USA; ^2^Department of Pediatrics, School of Medicine, Duke University, 905 S. LaSalle Street, Durham, NC 27710, USA

## Abstract

Better the drugs you know than the drugs you do not know. Drug repurposing is a promising, fast, and cost effective method that can overcome traditional de novo drug discovery and development challenges of targeting neuropsychiatric and other disorders. Drug discovery and development targeting neuropsychiatric disorders are complicated because of the limitations in understanding pathophysiological phenomena. In addition, traditional de novo drug discovery and development are risky, expensive, and time-consuming processes. One alternative approach, drug repurposing, has emerged taking advantage of off-target effects of the existing drugs. In order to identify new opportunities for the existing drugs, it is essential for us to understand the mechanisms of action of drugs, both biologically and pharmacologically. By doing this, drug repurposing would be a more effective method to develop drugs against neuropsychiatric and other disorders. Here, we review the difficulties in drug discovery and development in neuropsychiatric disorders and the extent and perspectives of drug repurposing.

## 1. Introduction 

The principle of “polypharmacology” (i.e., one drug, multiple hits, or off-target effects) has been understood since the advent of drug discovery. Traditionally, the goal of drug discovery and development was to identify the potential therapeutic agents using a one drug for one target model, suggesting that high selectivity (and/or affinity) would maximize efficacy and minimize side effects. In a series of efforts to identify such specific compounds (like using high throughput screening), there was a problem that a vast majority of compounds mediated unexpected and often undesired effects. The concept of polypharmacology emerged from this observation (i.e., drug promiscuity); however, polypharmacology should be distinguished from drug promiscuity. In our definition, drug promiscuity represents either good or bad effects mediated by compounds binding to both therapeutic and nontherapeutic targets, whereas polypharmacology represents beneficial effects mediated by compounds binding to multiple therapeutic targets. Various drug classes such as selective serotonin reuptake inhibitors [1, 2], antipsychotic [3], cholinesterase inhibitors [4], and thrombolytic agents [5] show polypharmacological features. In addition, amantadine was initially developed for influenza; however, after redirection, it is useful for Parkinson's disease [6, 7]. Zidovudine was intended to cancer treatment, and now it is redirected to targeting HIV/AIDS [8–10]. Additional, but well-known example is Viagra (Sildenafil) that was intended to antianginal medication but redirected to penile erections [11]. This growing evidence is against the simplicity of Ehrlich's “magic bullet concept” and it redirects our attention from the one drug-multiple target model to multiple mechanisms of action. Since every drug is able to hit multiple targets with and without our sense and knowledge [12], pursuing multiple targets for drugs should be accompanied by addressing a fundamental question: whether promiscuous drugs are able to contribute to their clinical efficacy out of the original scopes. A direct application of polypharmacology is drug repurposing which is also referred to as drug repositioning, drug reprofiling, and therapeutic switching. Generally, drug repurposing refers to a reinvestigation of existing drugs for new therapeutic interventions [13–17]. However, drug repurposing does not have to narrow down to take advantage of the off-target effects of the existing drugs as discussed later. In order to expand our knowledge and drug potentials, drug repurposing is a very productive method in drug discovery and development. It is useful for identifying and classifying drugs based on their actions to multiple therapeutic targets (i.e., leading to better efficacy and/or safety) or their action to nontherapeutic targets (i.e., leading to adverse effects). Drug repurposing can reduce the cost and risk intrinsic to drug discovery and development. This is especially valid, regarding the targeting of neurological and psychiatric disorders due to the complexity in their etiology and pathology. In this review, we will discuss the difficulties of the drug discovery and the development process with respect to neuropsychiatric disorders and the extent of drug repurposing as an alternative approach in drug discovery and development.

## 2. Challenges in Clinical Development for Neuropsychiatric Disorders

Due to the great progress and development of modern technology, our understanding of biological, physiological, and metabolic processes has advanced tremendously. However, we still face many challenges in drug discovery and development targeting neuropsychiatric disorders. There are four primary reasons why it is difficult to develop therapeutic agents against neuropsychiatric disorders: (1) CNS disorders have a complex etiology (heterogeneity; gene to environment), (2) limitations of understanding pathophysiology in neuropsychiatric disorders, (3) lack of appropriate biomarkers and/or molecular targets, and (4) lack of appropriate animal models. The etiological complexity of CNS disorders (e.g., schizophrenia) stems from multiple genetic and environmental factors [18, 19], sequentially giving rise to the other three problem categories mentioned above. Since the etiological complexity restricts our understanding of pathophysiology of CNS disorders, it is difficult to identify and/or characterize appropriate biomarkers and/or molecular targets. As a result, it is difficult to evaluate the mechanism(s) of action of therapeutic agents [20, 21]. In addition, their pharmacological manipulations targeting appropriate markers become risky. The absence of biomarkers and/or molecular targets prevents us from producing appropriate animal models that are genetically manipulated to recapitulate human disease conditions [22]. The clinical ineffectiveness of therapeutic agents may also occur due to the potential disagreements with the biological basis in the animal models and the mechanism(s) of action of therapeutic agents [23].

## 3. Promiscuity of Drug-Target Interactions

**Figure 1:**
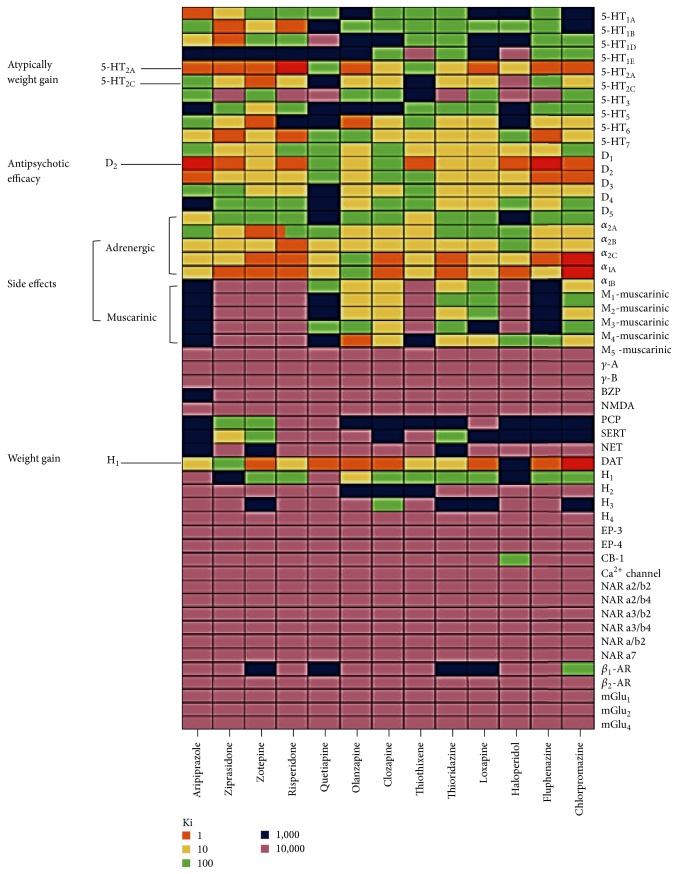
Polypharmacological profiles of antipsychotic drugs. Receptoromic screening identified multiple molecular targets for several antipsychotic drugs. In particulr, clozapine has the high affinity (Ki) to 5-HT serotonin receptors (5-HT2A, 5-HT2C, 5-HT6, and 5-HT7), dopamine receptor (D4), muscarinic receptors, (M1, M2, M3, M4, and M5), adrenergic receptors (*α*-1 and *α*-2), and other aminergic receptors. Other antipsychotic drugs also interact with multiple targets. More information can be found at NIMH PDSP database (http://pdspdb.unc.edu/pdspWeb/?site=kidb) (reprinted with permission from Nature Publishing Group).

Due to the complex nature of neuropsychiatric disorders (e.g., schizophrenia, depression, anxiety disorders, insomnia, migraine headaches, chronic pain and seizure disorders, and/or other complex mental disorders), the goal of therapeutic intervention is daunting. Although our knowledge and understanding of their pathophysiological phenomena have been greatly advanced and a series of efforts in CNS drug discovery and development adapted cutting edge technology of preclinical research and development, the discovery of specific CNS agents was not very successful and even depended on serendipity. This may be partially due to promiscuity of drug interactions. Multiple aspects (e.g., drugs and their target selectivity and affinity versus drugs' molecular targets and their mediated signaling pathways) must be considered in clinical drug development for neuropsychiatric disorders. Since the first introduction of the serotonin reuptake inhibitors (SSRIs, zimelidine, discovered in 1971 [24, 25]) and atypical antipsychotic agents (clozapine, discovered in 1958 [26]), the five most frequently prescribed CNS agents (olanzapine, quetiapine, risperidone, sertraline, and venlafaxine) share common mechanisms of action with the first two drugs (zimelidine and clozapine). Sharing mechanisms of action implicates that at least CNS agents may have common molecular targets or alter target-mediated signaling pathway(s) in one way or another. Furthermore, they may have marginal therapeutic effects on most of the common neuropsychiatric disorders. Indeed, about 70 compounds with common targets (serotonin receptors and dopamine receptors) are under development or in current use for treating schizophrenia and about 30 drugs aim at multiple therapeutic targets in schizophrenia [27, 28]. This is because compounds that aimed at one target can interact with others, supported by the golden standard of clozapine that it has polypharmacological profile with high affinity for several receptors as shown in Figure 1. These multiple actions of clozapine to multiple targets lead to a well-defined pharmacological signature in schizophrenia and related disorders in agreement with possible molecular targets in schizophrenia highlighted in Table 1 [29–31]. However, for example, after several trials of discovering and developing novel therapeutics like clozapine, none of the new compounds showed unique effectiveness compared with clozapine [32]. The promiscuity of drug-target interactions sometimes stems from a similarity; therapeutic and nontherapeutic molecular targets share high homology. Many molecular targets display their sequence similarity (and thus presumably similar conformation) and drugs themselves show structural similarity. For example, therapeutic agents targeting aminergic G-protein coupled receptors (GPCRs) are more promiscuous than other drug classes in spite of a diversity of GPCRs [33–36]. The similar moiety among drugs stems from the designated advantages over the existing drugs that aim at known molecular targets and/or are already effective. These drug classes can obtain polypharmacological profiles. Defining their actions to multiple targets (therapeutic targets versus nontherapeutic targets) makes gaining new opportunities in CNS agent discovery in the current efforts of clinical development for neuropsychiatric disorders difficult. Thus, the increase in promiscuity of drug-target interactions makes the discovery of CNS agents more complex.

## 4. Other Aspects in Drug-Target Interactions

We also should consider some other aspects that can provide us with the insights into the scope of CNS agent discovery. First two important aspects are functional selectivity and allosterism. Functional selectivity is defined as unique signaling pathways which are ligand-mediated. The ligand can be agonist or antagonist. They can modulate different signaling pathways through a single GPCR, resulting in different biological and/or physiological processes, depending on which pathway is activated [37–39]. Recently, Allen et al. studied the scaffold-based novel *β*-arrestin-biased ligand aripiprazole. A structure–functional–selectivity relationship (SFSR) of these novel compounds revealed distinct *β*-arrestin-bias toward the dopamine D2 receptor (D2R) [40]. Moreover, these unique D2R *β*-arrestin–biased agonists displayed atypical antipsychotic drug-like activities* in vivo* [41, 42]. Thus, it is often possible that drugs which may be thought to be nonselective to a particular target can actually interact with that target. However, due to the complexity of phenomena involved in biased signaling, the complete picture of the interactions between a receptor and a ligand can be difficult to access [38, 43, 44]. In addition, the results from inappropriate assays methodologically may provide precarious insights into their functional and/or molecular signatures, misdirecting researchers in the process of CNS drug discovery. However, the use of chimeric and/or promiscuous G proteins may partially overcome some of the potential difficulties of assessing drug-target interactions [45, 46] although considerable challenges remain. Allosteric modulation is the positively or negatively synergic effects on target function that are the result of a ligand binding to the binding site (allosteric) other than orthosteric binding site. Based on their binding effects, these ligand molecules are categorized as being positive of negative allosteric modulators. While the allosteric binding site does not accommodate endogenous ligands, allosteric modulators contribute to biological and physiological processes. Although the positive allosteric modulators do not activate receptors, they can enhance receptor-mediated activity at the presence of endogenous ligand. Since advantages over allosteric modulation are recently recognized in drug discovery and development, allosteric modulators appear to be more favorable therapeutic agents [47, 48]. The benzodiazepines are well known as allosteric modulators for GABA receptors which are in clinical use. However, pursuing allosteric modulators cannot be a panacea in CNS agent discovery because the presence of an orthosteric ligand is required for identifying allosteric modulators. This issue specifically applies to the targeting orphan GPCRs. There are additional problems in pursuing allosteric modulators. Allosteric binding sites are not evolutionarily conserved [48, 49], suggesting that they may be more diverse than orthosteric binding sites. Thus, it is possible that newly designed allosteric modulators can be more selective. However, this diversity and selectivity may cause other issues. For example, low evolutionary pressure of allosteric binding sites may result in many differences of receptor structure between species, which can make it difficult to employ* in vivo* animal models. The selectivity of allosteric modulators may not be verified because they bind to allosteric sites for their own targets and they may bind to orthosteric sites for nontargets. Then, allosteric modulator-dependent signaling pathways could lead to adverse side effects [50, 51]. The divergence of molecular targets also affects the efforts of CNS agent discovery. Many molecular targets have differential expression patterns and/or undergo posttranscriptional and/or posttranslational modifications. Such modifications and/or differential expression have significant effects on their confirmation and functions and eventually alter the properties of potential molecular targets [52–54]. It may be more difficult to identify and characterize drug-target interactions due to their divergence. For example, copy-number variations (CNVs) are recognized in risk for human diseases like schizophrenia [55–57], suggesting that some molecular targets among patients can be variable [58]; as a result, different or more promiscuous therapeutic agents are needed to aim at these variants or personalized medications can be an alternative solution. Receptor dimerization or oligomerization may contribute to the divergence of molecular targets, which is another challenge for CNS agent discovery. Even though the details of such mechanisms remain elusive, the receptor complex that mediates different signaling pathways may be important in the effectiveness of some CNS therapeutic agents [59, 60]. Designed multiple ligands (DMLs), which are the linked two therapeutic compounds, may be useful for manipulating receptor functions in targeting receptor complexes or two different receptors [60]. However, it may not be guaranteed whether DMLs have polypharmacological profile. Taken together, due to the complex nature of CNS disorders and challenges in basic and clinical researches, it is not surprising that we still face obstacle to the prospect of developing novel therapeutics with novel mechanisms. To accomplish major breakthrough, major challenges should be addressed to gain greater insight into the complexity of CNS disorders. In the next section, we will discuss an alternative approach (drug repurposing) in drug discovery and development in part to overcome the current drug discovery issues in neuropsychiatric disorders.

## 5. Drug Repurposing: Visit Your Drug Recycling Bins 

As discussed above, many hurdles still exist to develop CNS therapeutic agents, resulting in the fact that drug pipelines have gradually run out. The current approaches and methodology (e.g., high throughput screening and structure-based drug design) may not be an ideal solution for developing CNS agents because they are not cost and time-effective [61–64]. In addition, despite the extensive efforts of drug development, several drugs were withdrawn from the market due to severe side effects. For example, thalidomide was thought to be very safe [65]; however, it was withdrawn due to teratogenic effects (although it was appreciated on the cancer therapy market later). Thus, alternative approaches are necessary for overcoming such challenges. In agreement with the concept of Sir James Black, “the best way to discovery a new drug is start with an existing drug” [66], one of the alternatives, but a very attractive approach is drug repurposing that is a direct application of polypharmacological features of drugs in drug discovery and development against neuropsychiatric disorders [67–69]. Due to complex etiology of neuropsychiatric disorders, multiple therapeutic approaches may be essential so that polypharmacological approaches are well suited. A compound hitting multiple targets may have improved efficacy and/or safety in the view of therapeutic intervention for neuropsychiatric disorders. Different levels of polypharmacology could lead to better efficacy and/or safety or adverse effects. These differences in effectiveness may be due to the interaction of a compound with different therapeutic and/or nontherapeutic targets and/or dose dependence [70]. As mentioned earlier, polypharmacology-based drug repurposing refers to the reinvestigation of existing drugs for novel therapeutic purpose [13–17]. The existing drugs can be currently approved drugs or even “failed” compounds that their original scopes have already been identified. Failed compounds indicate abandoned drugs due to the fact that there is no clinical effectiveness even though the original scopes (e.g., initial targets in symptom/disorder) might be appropriate. Since it is highly possible that every drug displays off-target effects, the concept of a magic bullet (one drug, one target) is too simple and therefore archaic. Indeed, one drug can interact with diverse targets (average 6–13 multiple targets as predicted, [36]) and lead to multiple side effects. It does not matter if these effects are good or bad, however, as those side effects allow us to see different angles of drug usages [71–73]. Thus, polypharmacological profiling of these drugs against any druggable targets is greatly beneficial to drug discovery and development in order to decrease cost and time and enhance therapeutic potentials. Repurposing can decrease the time line for drug discovery and development. From target discovery to market, it takes 10 to 17 years in traditional drug discovery and development, whereas the duration of drug discovery for repurposed drugs takes from 3 to 12 years [13]. In addition, known safety and proven bioavailability of the existing drugs allow for an acceleration of the developmental process, reducing failure risk [74], although traditionally drug repurposing depended on serendipity, indicating a lack of processes and information. Presently, there are a variety of innovative approaches (e.g., cheminformatics, bioinformatics, and drug-target network) which may be very useful for drug repurposing. There are several tools to recycle the existing drugs to identify new therapeutic intervention for them as Jin and Wong summarized [75]. In order to expand therapeutic indication of existing drugs successfully, we need to be able to integrate multidisciplinary information derived from diverse fields such as cheminformatics, bioinformatics, chemistry, and* in vivo* and* in vitro* pharmacological assays and clinical trials. Based on this profiling information of the drugs (e.g., their known targets, toxicity, safety, and bioavailability) and/or diseases of interest, we can begin to find success in implementing drug repurposing. Here, we will highlight several tools and methods for drug repurposing and discuss how they can contribute to CNS drug discovery.

## 6. NIHM PDSP Database and Receptoromics as Drug Repurposing Tool

**Figure 2:**
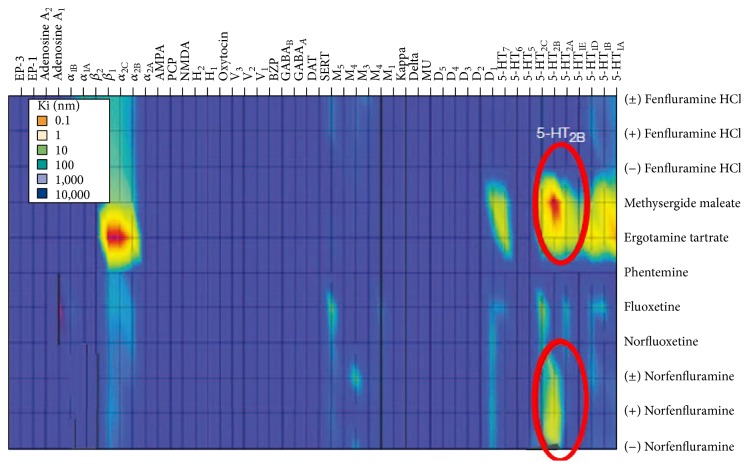
The molecular targets for cardiopulmonary-associated drugs. Recetoromic screening revealed the molecular targets implicated in fenfluramine. 5-HT2B serotonin receptor was identified as a molecular target for the norfenfluramine (a metabolite of fenfluramine), methylergonovine (a metabolite of the valvular heart disease- and pulmonary hypertension-associated drug methysergide), and dihydroergotamine (potentially associated with valvular heart disease and pulmonary hypertension). The drugs associated with cardio diseases also show high affinity to *α*-2B adrenergic receptors, whereas fluoxetine and the metabolite norfluoxetine do not. In this heat map, the affinity of the drugs is mapped by color gradient, that is, blue (lower affinity, higher Ki), red (higher affinity, lower Ki), and intermediated color (reprinted with permission from Nature Publishing Group).

As various tools and methods have been developed and refined, corresponded databases have been constructed. Two-pioneered approach was dubbed receptoromics [76–81] and the NIMH PDSP database (National Institute of Mental Health Psychoactive Drug Screening Program) was created [77, 82, 83]. Since receptoromic approach encompasses computational and physical screening tools, it is an ideal tool for drug repurposing. In one way of a computational tool (or* in silico* approach), it has been referred as a receptor structure-function basis to be able to screen compounds virtually. At the beginning, several GPCR molecular models were based on homology modeling with bovine rhodopsin [84, 85]. However, as more validated molecular structures are solved [86–91], compound libraries can be screened* in silico* against a virtual receptorome in order to identify potential drug and target interactions. In line with* in silico* approach, the drug information databases are also extremely useful. Such databases may facilitate finding of target-specific drugs, identification of lead compounds, and characterization of ligand's structural features. The publically available database, NIMH PDSP Ki was constructed from data obtained through high or medium throughput receptoromic screening; this database is continually expanding. This information provides valuable insights into drug repurposing. Another way of a physical screening tool has been referred to as phenotypic/target-based screening that is used for receptoromic profiling. It can help in the identification of molecular targets for endogenous ligands. The data from physical screening also comes from ligand binding screening, functional screening, and validation of molecular targets. By using such computational and physical readouts, many targets can be screened in parallel. In addition, we can identify possible therapeutic or adverse effects of the existing compounds by blindly screening them against multiple targets in a nonbiased way. As a result, significant discoveries have already been reported. For example, 5-HT2B serotonin receptors may serve as a molecular target responsible for fenfluramine-induced valvular heart disease as receptoromic screening indicated that norfenfluramine, via activating 5-HT2B serotonin receptors, was a potential agent responsible for valvular heart disease and pulmonary hypertension (Figure 2; Fen-phen [92–95]). Additionally, H1 histamine receptors may serve as a molecular target responsible for weight gain in patients taking atypical antipsychotics [96]. Kappa-1 opioid receptors may serve as a molecular target on the hallucinogenic effects of salvinorin A [97]. Thus, receptoromic screening of drugs can provide insight for repurposing drugs because this nonbiased and parallel screening allows us to discover the molecular mechanisms responsible for and to predict drug-mediated side effects.

## 7. Other Databases and Potential Tools for Drug Repurposing

In addition to the NIMH PDSP database, other chemical databases are available. For example, Ligand Expo [98], ZINC [99], and KEGG DRUG [100] databases integrate diverse information such as molecular pathways, binding experiments, and drug targets. One of the large public databases, PubChem, containing the results from many screens and assays [101, 102], can also facilitate drug repurposing. The PubChem database includes 61 psychiatric drugs that were assessed in a number of different bioassays, resulting in that those drugs were identified as “active” in multiple bioassays. These data can be a fundamental resource of drug repurposing. The European College of Neuropsychopharmacology (ECNP) also provides valuable tools (e.g., ECNP Medicines chest) to help clinical researchers obtain access to pharmacological tools important for the pursuit of their studies (https://www.ecnp.eu/). Additionally, Pouliot and colleagues already used PubChem bioassay data for predicting adverse drug reactions (ADRs) [103]. Growing availability of such databases enables various tools to be applied to drug repurposing. For example, Keiser et al. reported Similarity Ensemble Approach (SEA) to categorize molecular drug targets through the similarity of their known molecular ligands [104–106]. This similarity based drug-target interaction database is further used for predicting novel drug-target interactions. Practically, out of 30 predictions, the authors confirmed 23 in experimental assays [105]. Drug-target binary association was created to build bipartite graphs, indicating the linkage between FDA approved drugs and target proteins [107]. Side effect similarity allowed inferring if two drugs share their targets [108]. Additionally, in order to predict the potential off-target effects for any given drug, ligand target interaction/molecular networks [109, 110] were reported. The comparative toxicogenomics database (CTD) was developed to provide insight into complex chemical, gene, and protein interaction [111]. Adverse reactions of drugs have been mapped by bioinformatic mining of approved drug information. Based on 7,684 approved drug labels, they mapped the adverse reactions corresponding to 988 unique drugs onto 174 side effects [112, 113]. Weighted network based inference methods enable us to predict chemical and protein interactions [114]. Recently, a self-organizing map based prediction of drug equivalence relationships (SPiDER) was developed to identify targets, both for known drugs and computer-generated molecular scaffolds [115]. Genome-based drug reposition is additional strength regarding the potential tools for drug repurposing [15, 116]. The integrative genome-wide metrics can provide insightful scalar theories of massive biological information. These drug repurposing tools and multiple databases can be applied to identify new therapeutic interventions for the existing drugs and can accelerate CNS drug discovery.

## 8. Conclusions 

Despite the exponential growth of advanced technology and the resultant molecular database, we still do not fully understand the signaling pathways and molecular mechanisms behind many disease states, especially neuropsychiatric disorders. This lack of knowledge makes repurposing drugs difficult and that is a direct application of polypharmacology. Drug repurposing does not eliminate the risk of compound development; however, it reduces the risk of the lack of compound development. There are already several CNS therapeutic drugs which have been repurposed (summarized in Table 2). Moreover, another application of polypharmacology could be designed for multitarget ligands [67, 117]. If we designed ligands against profiles of multiple therapeutic targets, it would accelerate CNS drug discovery and enhance drug repurposing [60]. Indeed, Apsel and coworkers reported compounds that inhibit both tyrosine kinases and phosphatidylinositol-3-OH kinases [118]. This work is a successful example of rational design of multitarget ligands in the human kinome context. In addition, Besnard et al. successfully launched a new approach for the automated design of ligands with polypharmacological profiles [119]. Although it will be a challenge to rationally design multitarget ligands with therapeutic benefits, advancing the automated design of ligands and other areas in developing, optimizing, and validating target combinations will be the key in the future for rational design of drugs with polypharmacological profiles. The potential advantages in computer-aided drug repurposing enable us to prioritize CNS drug discovery. In addition, experimental approaches such as phenotypic screening are investigated for drug repurposing [52]. Therefore, the application of these combinatorial approaches to neuropsychiatric disorders will not only provide great opportunities in drug discovery and development but also lead to the identification of nontherapeutic targets that can cause adverse effects.

## Figures and Tables

**Table 1:** Potential molecular targets in schizophrenia.

Molecular targets proposed for schizophrenia	
Target family	Drug actions

Dopamine receptors	D1 agonists
	D2 antagonists
	D4 antagonists
	D4 agonists

Serotonin receptors	5-HT1A agonists
	5-HT1A antagonists
	5-HT2A antagonists
	5-HT4 agonists
	5-HT6 agonists
	5-HT7 agonists

Muscarinic receptors	M1 agonists
	M4 agonists
	M5 antagonists

GABA receptors	GABA.a (*α*2) agonists
	GABA.a (*α*5) antagonists

Adrenergic receptors	*α*2 adrenergic antagonists

Glutamate receptors	Glycine transporter inhibitors
	mGluR2/3 agonists
	mGluR5 agonists
	NMDA enhancers

Others	Nicotinic *α*7 agonists
	Nicotinic *α*4*β*2 agonists
	Ampakines
	COMT inhibitors

**Table 2:** Some examples of repurposed drugs for neuropsychiatric disorders.

Drugs (alphabetic order)	Actions/classes	First intervention	New intervention	References
Amantadine	Anticholinergic-like agent	Influenza	Parkinson's disease, ADHD	[6, 7]
Amphotericin B	NSAID^*∗*^	Antifungal	Bipolar disorder	[120]
Arbaclofen	GABA agonist	Cerebral palsy	Fragile X syndrome	[116, 121–123]
Atomoxetine	NSRI^*∗∗*^	Parkinson's diseases	ADHD	[124]
Dexmecamylamine	Nicotinic receptor modulator	Hypertension	Depression	[125, 126]
Galantamine	Acetylcholinesterase inhibitor	Polio, paralysis	Alzheimer's disease	[127]
Mecamylamine	Nicotinic receptor antagonist	Hypertension	ADHD Depression	[128–131]
Mifepristone	Glucocorticoid receptor type II antagonist	Pregnancy termination	Psychotic major depression, Cushing's syndrome	[132–135]
Ropinirole	D2 agonist	Hypertension	Parkinson's disease, idiopathic restless leg syndrome	[136–138]
Tamoxifen	Estrogen receptor	Breast tumor	Bipolar disorder Mania	[139]
Valsartan	Angiotensin receptor blocker	Hypertension	Alzheimer's disease	[140]

^*∗*^NSAID is nonsteroidal anti-inflammatory drug.

^*∗∗*^NSRI is norepinephrine-selective reuptake inhibitor.

## Abbreviations

5-HT1A: 5-Hydroxytryptamine (serotonin) receptors
D2: Dopamine receptors
*α*2AR: *α* Adrenergic receptors
*β*2-AR: *β*-Adrenergic receptors
M1: Muscarinic receptors
*γ*-A, B: *γ*-Aminobutyric acid receptors
BZP: Benzodiazepines
NMDAR: N-Methyl-D-aspartate receptor
AMPAR: *α*-Amino-3-hydroxy-5-methyl-4-isoxazolepropionic acid receptor
PCP: Phencyclidine
SERT: Serotonin transporter
NET: Norepinephrine transporter
DAT: Dopamine transporter
H1R: Histamine receptors
EP-3, 4: Prostaglandin receptors
CB-1: Cannabinoid receptors
NAR: Nicotinic acetylcholine receptors
mGluR: Metabotropic glutamate receptors
V1: Vasopressin receptors
*κ*, *δ*, *μ*: Opioid receptors (kappa, delta, and mu).
